# Ocular Findings Among Patients With Vitamin D Deficiency

**DOI:** 10.7759/cureus.15159

**Published:** 2021-05-21

**Authors:** Hatice Daldal, Ayla Gokmen Salici

**Affiliations:** 1 Department of Ophthalmology, Usak University Faculty of Medicine, Usak, TUR; 2 Department of Internal Medicine, Usak University Training and Research Hospital, Usak, TUR

**Keywords:** vitamin d deficiency, ocular findings, retinal nerve fiber layer thickness, nasal quadrant, central macular thickness

## Abstract

Objective

The aim of this study was to evaluate the ocular findings in patients with low vitamin D levels.

Methods

All patients who attended the Internal Medicine Clinic between March 2018 and February 2020 with vitamin low D levels but had been untreated for the same were included in our study. The exclusion criteria were as follows:history of intraocular surgery, trauma, steroid use, secondary glaucoma, and history of rheumatologic diseases. The patients were classified into three groups: group 1 had severe deficiency with vitamin D levels below 10 µg/L; group 2 had vitamin deficiency with levels of 10-20 µg/L; and group 3 had vitamin D insufficiency with levels of 20-30 µg/L. A comparison among groups was performed in terms of intraocular pressure (IOP), retinal nerve fiber layer (RNFL) thickness, central macular thickness (CMT), dry eyes, cataract, glaucoma, macular degeneration, and refractive error. The evaluation of statistical data was performed with the SPSS Statistics software version 22 (IBM, Armonk, NY).

Results

There were a total of 98 patients and 196 eyes, who were classified into three groups. There were 41 patients in group 1, 45 in group 2, and 12 in group 3. Groups were similar in terms of age (p=0.25) and gender (p=0.46). The average age among the cohort was 51 ± 13.08 years; 65 (66.3%) of the patients were female and 33 (33.7%) were male. There was no statistically significant difference in terms of IOP (p=0.55), dry eyes (p=0.35), cataract (p=0.22), glaucoma (p=0.50), macular degeneration (p=0.64), and refractive error (p=0.46) among the groups. There was a statistically significant difference in CMT between group 1 and other groups (p=0.002 and p=0.002, respectively). Also, there was a statistically significant difference in RNFL thickness between group 1 and group 2 (p=0.01). When compared in terms of quadrants, a significant difference was found only with regard to the nasal quadrant.

Conclusion

Based on our findings, lower levels of 25-hydroxyvitamin D might be related to thinning in CMT. Regarding RNFL thickness, while there was a significant difference between groups 1 and 2, there was no difference between groups 1 and 3, and hence the association between lower levels of 25-hydroxyvitamin D and thinning in RNFL thickness could not be clearly established. Hence, we have assumed that lower levels of 25-hydroxyvitamin D might cause thinning in the macula and nasal quadrant of RNFL, and vitamin D deficiency might affect the nasal quadrant of RNFL primarily. Further long-term studies with a larger number of patients might clarify the relationship between vitamin D deficiency and the thinning in CMT, RNFL quadrants, and RNFL thickness.

## Introduction

More than half of the global population is thought to be at risk of vitamin D deficiency [1]. Vitamin D deficiency is also widespread in Turkey. A meta-analysis has revealed a high rate of vitamin D deficiency in Turkey, which varies from 58.9-66.6% [2,3]. Vitamin D deficiency can result in conditions such as rickets and osteomalacia [4,5]. Recently, vitamin D deficiency has been associated with many diseases such as cardiovascular disorders, metabolic syndrome, infectious and autoimmune conditions, as well as ocular diseases [6,7,8]. As per the classification based on 25-hydroxyvitamin D levels, individuals with levels over 30 ng/ml are considered to have sufficient vitamin D; however, levels of 20-30 ng/ml are classified as vitamin D insufficiency, levels below 20 ng/ml as deficiency of vitamin D, and those below 10 ng/ml are regarded as serious deficiency of vitamin D [9]. In this study, we aimed to evaluate ocular findings among patients with vitamin D deficiency (levels below 30 µg/L).

## Materials and methods

This was a prospective study involving untreated patients with low levels of 25-hydroxyvitamin D who attended the Internal Medicine Clinic between March 2018 and February 2020. Ethical approval from the Usak University Faculty of Medicine Ethics Committee was obtained before commencing the study. Patients with a history of previous intraocular surgery, trauma, steroid use, secondary glaucoma, rheumatological disease, and other systemic additional diseases such as diabetes mellitus and hypertension were excluded from the study. Most of the patients had been referred with complaints of malaise and fatigue, while some patients had myalgia, and some patients were asymptomatic volunteers.

The patients were divided into three groups. Group 1 consisted of patients with severe 25-hydroxyvitamin D deficiency (below 10 µg/L); group 2 comprised patients with 25-hydroxyvitamin D deficiency with levels between 10-20 µg/L; and group 3 consisted of patients with insufficiency of 25-hydroxyvitamin D with levels between 20-30 µg/L [9].

A comparative analysis was performed between the groups in terms of intraocular pressure (IOP), retinal nerve fiber layer (RNFL) thickness, central macular thickness (CMT), dry eyes, cataract, glaucoma, macular degeneration, and refractive error.

IOP was measured with an applanation tonometer. RNFL thickness and CMT values were measured using spectral-domain optical coherence tomography (OCT) (Cirrus HD-OCT 4000; Carl Zeiss Meditec, Jena, Germany). OCT measurements were done bilaterally. Perimetry was applied to patients who had a decrease in RNFL thickness and increase in cup/disc ratio and IOP for the diagnosis of glaucoma, in addition to routine ocular examination and evaluation of cup/disc ratio. The presence of dry eyes was evaluated by the Schirmer test and tear film break-up time test.

Statistical analysis was performed with the SPSS Statistics software version 22 (IBM, Armonk, NY), by using chi-square and Kruskal-Wallis tests. A p-value of <0.05 was considered to be statistically significant.

## Results

The study involved 196 eyes of 98 patients: 41 in group 1, 45 in group 2, and 12 in group 3. The groups were similar in terms of age (p=0.25) and gender (p=0.46); 65 (66.3%) of the patients were female and 33 (33.7%) were male. The average age was 49.17 ± 13.00 years in group 1, 50.93 ± 12.35 years in group 2, and 53.16 ± 12.44 years in group 3. There was no statistically significant difference between the groups in terms of IOP (p=0.55), presence of dry eyes (p=0.35), cataract (p=0.22), glaucoma (p=0.50), macular degeneration (p=0.64), and refractive error (p=0.46) (Table 1).

However, there was a statistically significant difference in CMT between group 1 and other groups (243 ± 18.4 µ, 252 ± 17 µ, 256 ± 13.4 µ; p=0.002, p=0.002, respectively). There was no statistically significant difference in CMT between group 2 and group 3 (252 ± 17 µ, 256 ± 13.4 µ; p=0.30). There was a statistically significant difference in RNFL thickness between group 1 and group 2 (93.7 ± 9.89 µ, 91.4 ± 8.51 µ; p=0.01). When group 1 and group 3, and group 2 and group 3 were compared, there was no statistically significant difference in RNFL thickness (93.7 ± 9.89 µ, 91.4 ± 8.51 µ, 94.8 ± 7.47 µ; p>0,99, p=0.26, respectively) (Table 2).

When compared in terms of quadrants, there was a significant difference only with regard to the nasal quadrant: a significant difference was observed between groups 2 and 3 (mean nasal quadrant of RNFL was 68.53 µ in group 1, 67.72 µ in group 2, and 74.95 µ in group 3) (Table 3).

**Table 1:** Comparison between groups in terms of IOP, presence of dry eyes, cataract, glaucoma, macular degeneration, and refractive error SD: standard deviation; IOP: intraocular pressure

Variables	Group 1 (n=41)	Group 2 (n=45)	Group 3 (n=12)	P-value
IOP, mmHg (median ± SD)	13.30 ± 2.91	13.06 ± 2.50	12.45 ± 2.08	0,55
Dry eye, n (%)	14 (34.14%)	16 (35.55%)	4 (33.33%)	0.35
Cataract, n (%)	14 (34.14%)	15 (33.33%)	4 (33.33%)	0.22
Glaucoma, n (%)	0 (0%)	0 (0%)	0 (0%)	0.50
Macular degeneration, n (%)	4 (9.75%)	5 (11.11%)	1 (8.33%)	0.64
Refractive error, n (%)	Myopia: 9 (21.95%); hyperopia: 7 (17.07%); astigmatism: 14 (34.14%)	Myopia: 10 (22.22%); hyperopia: 7 (15.55%); astigmatism: 16 (35.55%)	Myopia: 3 (25%); hyperopia: 2 (16.66%); astigmatism: 4 (33.33%)	0.46

**Table 2:** Comparison between groups in terms of CMT and RNFL thickness *Statistically significant SD: standard deviation; CMT: central macular thickness; RNFL: retinal nerve fiber layer

Variables	Group 1 (n=41)	Group 2 (n=45)	Group 3 (n=12)	P-value (group1 vs. group 2/group 2 vs. group 3/group 1 vs. group 3)
CMT, µ (mean ± SD)	243 ± 18.4	252 ± 17	256 ± 13.4	0.002*/0.30/0.002*
RNFL thickness, µ (mean ± SD)	93.7 ± 9.89	91.4 ± 8.51	94.8 ± 7.47	0.01*/0.06/0.86

**Table 3:** Comparison between groups in terms of RNFL quadrants *Statistically significant SD: standard deviation; RNFL: retinal nerve fiber layer thickness

RNFL	Group 1 (n=41)	Group 2 (n=45)	Group 3 (n=12)	P-value (group1 vs. group 2/group 2 vs. group 3/group 1 vs. group 3)
Nasal, µ (mean ± SD)	68.53 ± 9.49	67.72 ± 9.81	74.95 ± 13.90	0.63/0.04*/0.11
Temporal, µ (mean ± SD)	65.34 ± 10.81	64.21 ± 11.03	67.00 ± 12.91	0.56/0.25/0.36
Superior, µ (mean ± SD)	118.73 ± 14.44	116.43 ± 15.64	117.79 ± 14.73	0.28/0.94/0.35
Inferior, µ (mean ± SD)	121.68 ± 17.54	117.21 ± 18.61	119.25 ± 23.11	0.17/0.44/0.30

## Discussion

Recent studies have revealed that vitamin D deficiency can lead to various ocular diseases. Those with low 25-hydroxyvitamin D levels have been associated with the risk of diseases involving dry eyes, diabetic retinopathy, myopia, age-related macular degeneration (AMD), whereas higher levels have been linked with decreased frequency of diseases. Also, vitamin D treatment has been experimentally shown to improve the conditions of those with diseases such as retinoblastoma, choroidal melanoma, retinal aging, ischemic retinopathy, diabetic retinopathy, autoimmune uveitis, corneal damage, and glaucoma [8].

There are studies that show that vitamin D affects eye cells. Initially, vitamin D-dependent calcium-binding protein was found in the human retina [10]. Afterward, vitamin D receptors (VDR) were detected in the cornea, lens, epithelia of the ciliary body, retinal pigment epithelia, corneal endothelium, ganglion cell layer, and photoreceptors with immunohistochemical staining [11]. VDRs are expressed in the retina and choroid by vascular endothelial cells [12]. Recently, vitamin D hydroxylase has been detected in the corneal epithelium, endothelium, sclera, non-pigmented ciliary epithelium, and retinal pigment epithelium, and these cells have been thought to play a part in vitamin D metabolism [13].

In a study by Tideman et al., consisting of a total of 2,666 children aged six years onwards, low 25-hydroxyvitamin D concentration in serum was found to be related to longer axial length and higher risk of myopia, regardless of the level of outdoor exposure [14]. In another study by Mutti, it was speculated that 25-hydroxyvitamin D might have a potential therapeutic effect in controlling the increase in myopia rates [15]. In a study by Yazar et al., 221 (23.4%) of 946 participants were found to have myopia. Individuals with myopia have been shown to have lower serum 25-hydroxyvitamin D concentrations compared to non-myopic participants. Low serum 25-hydroxyvitamin D levels have been associated with a higher risk of myopia (<50 vs. ≥50 nmol/L; p<0.001). The prevalence of myopia has been reported to be significantly higher in vitamin D-deficient individuals compared with patients who have sufficient amounts of vitamin D [16].

In a study by Choi et al., a total of 2,038 individuals aged 13-18 years were evaluated, and low 25-hydroxyvitamin D levels were found to be associated with the prevalence of myopia. This relationship has been reported to be more remarkable in adolescents with high myopia [17]. Some studies have indicated variations in the VDR as a potential risk factor for myopia development. The VDR gene is located near the loci found to be related to myopia (MYP-3). These results suggest that the VDR gene does not play a direct role in myopia development, but it can indirectly contribute to the risk caused by mechanical stress factors or growth factors, by way of its role in calcium homeostasis and the regulation of ciliary muscle function [18].

In our study, there was no significant difference between the groups in terms of myopia or other refraction errors. The reason for this is that all the patients included in the study had a 25-hydroxyvitamin D level below 30 µg/L, and there were no patients with levels above 30 ng/ml.

There are studies in the literature on AMD, which is one of the diseases linked with the deficiency of vitamin D. In a study consisting of a total of 96 patients, including 30 with late-stage AMD, 32 with early-stage AMD, and 34 normal controls, it was hypothesized that the deficiency of vitamin D might increase the risk of early and late AMD and might be related to subretinal fibrosis [19].

A diet rich in vitamin D has been shown to prevent or delay the progression to advanced AMD, particularly neovascularization [20]. High vitamin D levels are associated with a decreased risk of early or advanced stages of AMD in many studies. In a study by the Carotenoids in Age-related Eye Disease Study (CAREDS) group consisting of 1,313 people, the risk of early-stage AMD was found to be 48% less in women under 75 years of age with 25-hydroxyvitamin D levels above 30 ng/ml. This study suggests that 25-hydroxyvitamin D may have a preventive effect in early-stage AMD in those under 75 years of age [21].

In another study involving 7,752 people over 40 years of age, patients with 25-hydroxyvitamin D levels above 34 ng/ml and those with levels below 17 ng/ml were compared, and it was found that the risk of early-stage AMD was 36% and the risk of soft drusen was 24% less in individuals with 25-hydroxyvitamin D levels above 34 ng/ml [22]. In a study by Graffe et al., age, gender, comorbidity, cognitive changes, body mass index, mean arterial pressure, visual acuity, IOP, and serum Ca levels were evaluated as possible contradictory factors when the test was performed and corrected. Macular thickness was reported to be thinner in the group with low 25-hydroxyvitamin D levels compared to those with sufficient levels (232.9 ± 40.4 µ and 253.3 ± 32.1 µ, respectively) [23]. Similarly, in our study, a significant difference was detected between group 1 and other groups in terms of CMT (243 ± 18.4 µ, 252 ± 17 µ, 256 ± 13.4 µ). Hence, we hypothesize that lower levels of 25-hydroxyvitamin D might cause macular thinning.

In the study by Dikci et al. involving 31 pseudoexfoliation cases with glaucoma, 34 cases with pseudoexfoliation syndrome, and 43 controls, 25-hydroxyvitamin D levels details of glaucoma surgery were compared between the groups. There was no significant difference between 25-hydroxyvitamin D levels. Based on this finding, 25-hydroxyvitamin D level was thought to have no effect on the development of pseudoexfoliation glaucoma/syndrome [24]. In another study, deficiency of vitamin D and the presence of BsmI 'B' allele and TaqI 't' allele were found to be related risk factors in glaucoma development [25]. In our study, glaucoma was not detected in any of the patients.

Some studies have shown that the deficiency of vitamin D is associated with cataracts [26,27]. In our study, cataracts were detected in 34.14% of the patients in group 1, 33.33% in group 2, and 33.33% in group 3, and no difference was found between the groups. In a study by Demirci et al., it was determined that the deficiency of vitamin D is related to tear hyperosmolarity and tear film dysfunction and might cause susceptibility to dry eyes [28]. In the study by Kizilgul et al., replacement therapy was shown to improve tear osmolarity in patients who have vitamin D deficiency [29]. In our study, dry eyes were detected in 34.14% of the patients in group 1, 35.55% in group 2, and 33.33% in group 3, and no significant difference was detected between the groups.

In the study by Uro et al. involving older patients, one group comprising patients with vitamin D deficiency (<25 nmol/L) and those with sufficient levels (>25 nmol/L) were compared. It was determined that the deficiency of vitamin D in older patients is associated with decreased mean ganglion cell complex thickness before the loss of RNFL; there was no difference in the mean RNFL thickness between these two groups [30].

In our study, vitamin D deficiency's effect on RNFL quadrants was evaluated differently from other studies, and a difference in the thickness of the RNFL nasal quadrant was detected between the vitamin D-deficient group and the vitamin D-insufficient group. Although CMT was thinner in group 1 when compared to other groups, RNFL was greater in group 1 compared to group 2 but similar to group 3. Thus, we could not establish a reliable association between 25-hydroxyvitamin D levels and RNFL thickness. Studies with a larger cohort of patients are required to better evaluate vitamin D deficiency's effect on RNFL quadrants.

The main limitation of our study was the absence of a control group. Unfortunately, during the study period, none of the volunteers and patients who attended the Internal Medicine Clinic had 25-hydroxyvitamin D levels >30 ng/mL. This could be attributed to the high rate of vitamin D deficiency in our country, as shown in the previously mentioned meta-analysis [3]. Another limitation is the small number of patients in group 3. In light of these limitations, we believe that further long-term studies with a larger number of patients are required to provide more clarity on the relationship between vitamin D and the thinning in CMT, RNFL quadrants, and RNFL thickness.

This article was previously presented as an oral presentation at the Third International Hippocrates Congress on Medical and Health Sciences, on March 6-7, 2020 in Ankara, Turkey.

## Conclusions

Based on our findings, the lower levels of 25-hydroxyvitamin D may be related to the thinning in CMT. In terms of RNFL thickness, while there was a significant difference between the severe vitamin d-deficient group (group 1) and the vitamin d-deficient group (group 2), there was no difference between the severe vitamin d-deficient group (group 1) and the vitamin d-insufficient group (group 3), and hence the association between lower levels of 25-hydroxyvitamin D and the thinning in RNFL thickness could not be conclusively established. When compared in terms of quadrants, there was a significant difference only with regard to the nasal quadrant between the vitamin D-deficient group (group 2) and the vitamin D-insufficient group (group 3). In light of these findings, we have hypothesized that lower levels of 25-hydroxyvitamin D might cause thinning in the macula and nasal quadrant of RNFL and vitamin D deficiency might affect the nasal quadrant of RNFL primarily.
